# Serum Saturated Fatty Acids including Very Long-Chain Saturated Fatty Acids and Colorectal Cancer Risk among Chinese Population

**DOI:** 10.3390/nu15081917

**Published:** 2023-04-15

**Authors:** Qixin Wu, Dandan Shi, Ting Dong, Zhuolin Zhang, Qingjian Ou, Yujing Fang, Caixia Zhang

**Affiliations:** 1 Department of Epidemiology, School of Public Health, Sun Yat-sen University, Guangzhou 510080, China; wuqx8@mail2.sysu.edu.cn (Q.W.); shidd3@mail2.sysu.edu.cn (D.S.); dongt23@mail2.sysu.edu.cn (T.D.); zhangzhlin7@mail2.sysu.edu.cn (Z.Z.); 2 Department of Experimental Research, Sun Yat-sen University Cancer Centre, State Key Laboratory of Oncology in South China, Collaborative Innovation Centre for Cancer Medicine, Guangzhou 510060, China; ouqj@sysucc.org.cn

**Keywords:** saturated fatty acids, very long-chain saturated fatty acids, colorectal cancer, serum, risk

## Abstract

The association between circulating saturated fatty acids (SFAs) including very long-chain SFAs (VLCSFAs) and colorectal cancer (CRC) risk has not been clearly established. To investigate the association between serum SFAs and CRC risk in Chinese population, 680 CRC cases and 680 sex and age-matched (5-year interval) controls were recruited in our study. Serum levels of SFAs were detected by gas chromatography. Unconditional logistic regression models were used to estimate odds ratios (ORs) and 95% confidence intervals (CIs) for the association between serum SFAs and CRC risk. Results showed that total SFAs were positively associated with the risk of CRC (adjusted OR _quartile 4 vs. 1_ = 2.64, 95%CI: 1.47–4.74). However, VLCSFAs were inversely associated with CRC risk (adjusted OR _quartile 4 vs. 1_ = 0.51, 95%CI: 0.36–0.72). Specifically, lauric acid, myristic acid, palmitic acid, heptadecanoic acid, and arachidic acid were positively associated with CRC risk, while behenic acid and lignoceric acid were inversely associated with CRC risk. This study indicates that higher levels of total serum SFAs and lower levels of serum VLCSFAs were associated with an increased risk of CRC in Chinese population. To reduce the risk of CRC, we recommend reducing the intake of foods containing palmitic acid and heptadecanoic acid such as animal products and dairy products, and moderately increasing the intake of foods containing VLCSFAs such as peanuts and canola oil.

## 1. Introduction

Colorectal cancer (CRC) is the third most prevalent malignancy worldwide, with more than 1.9 million new CRC cases occurring in 2020, representing about one-tenth of cancer cases [1]. The roles of dietary fats and fatty acids in CRC risk have always been issues of interest [2,3]. Recent research revealed that fatty acids play an important role in the development of cancer [4]. Saturated fatty acids (SFAs) have been shown to promote CRC by affecting cancer cells from the susceptibility of cellular membranes to lipid peroxidation [5]. However, SFAs can be obtained not only through dietary intakes, but also synthesized in vivo [6]. The primary product of de novo lipogenesis is palmitic acid (C16:0). Additionally, dietary sources of SFAs are also different. Lauric acid (C12:0) and myristic acid (C14:0) are derived from vegetable oils [7]. Palmitic acid (C16:0) and stearic acid (C18:0) are found mainly in animal products and tropical oils [7]. Heptadecanoic acid (C17:0) is found in dairy products [8], whereas arachidic acid (C20:0), behenic acid (C22:0), and lignoceric acid (C24:0) are found mainly in peanuts, macadamia nuts, and canola oil. Therefore, circulating SFAs combining endogenous and exogenous SFAs can be better used to examine the association between SFAs and diseases [8].

Although circulating SFAs have been suggested to play a promoting role in the development of breast cancer [9,10], prostate cancer [11,12], and hepatocellular carcinoma [13], the association between circulating SFAs and CRC has not been clearly established [2,3,14,15,16]. Some [2,15,16], but not all [3,14], studies reported that total plasma phospholipid or erythrocyte SFAs were positively associated with CRC risk. Moreover, various chain lengths of SFAs may affect CRC differently, as they may differ in dietary sources, endogenous metabolism, and biological activity. Previous studies have examined the association between individual SFAs and CRC [2,3,15,16]. However, the studied individual SFAs were incomplete, and the results were conflicting.

Very long-chain saturated fatty acids (VLCSFAs) have 20 or more carbons. Recent research suggests that VLCSFAs have a deep influence on inflammation resolution and ceramide-induced apoptosis, which could affect tumour growth [17,18]. Some studies found that circulating VLCSFAs were inversely associated with cardiovascular diseases [19,20], diabetes [21], and non-Hodgkin lymphoma [22]. However, no published studies have investigated the association between circulating VLCSFAs and CRC risk.

Most previous studies investigating the association between circulating SFAs and CRC risk were conducted in Western countries or developed countries, where dietary habits differ from Chinese population [2,3,23]. To our knowledge, no study has reported the association between circulating SFAs including VLCSFAs and CRC risk in China. Therefore, to explore different serum levels of SFAs associated with CRC risk, we examined the association of serum concentrations of SFAs including VLCSFAs with CRC risk in Chinese population. We hypothesize that higher levels of serum SFAs are positively associated with CRC risk, while high levels of serum VLCSFAs are inversely associated with CRC risk. The results of our study might help to provide insights into the roles of individual SFAs in CRC risk and provide research evidence for the targeted cancer prevention strategies.

## 2. Materials and Methods

### 2.1. Study Design and Study Sample

This case-control study was conducted as part of a research program on dietary and lifestyle factors related to CRC in China. The study design has been described previously [24,25]. Cases with histologically confirmed CRC diagnosis within 3 months have been recruited by Sun Yat-sen University Cancer Centre, Guangzhou, China, from July 2010 to May 2021. Patients without a prior diagnosis of cancer, aged 30–75 years, Guangdong natives, or who have lived in Guangdong Province for more than 5 years were invited to participate in the study. We excluded participants who could not understand or communicate in Mandarin or Cantonese. Totally, 3174 cases were surveyed, and 2833 eligible cases finished the interview, with a response rate of 89.26%. In this study, we randomly selected 680 blood samples from these cases for the measurement of fatty acids.

Controls were selected from two groups. One group was recruited from the Departments of Otorhinolaryngology, Vascular Surgery, and Plastic Surgery in the First Affiliated Hospital, Sun Yat-sen University, during the period of case collection for CRC. They mainly had sudden deafness, chronic otitis media, chronic sinusitis, polyp of the vocal cord, trigeminal neuralgia, varicose veins, orthopaedics, and facial paralysis. There was no evidence that the above diseases were related to dietary causes, so we selected the controls from these departments. The other group was enrolled from residents of the same community via advertisements, written invitations, or referrals from participants. Control subjects had the same inclusion and exclusion criteria as the cases, with the exception that they had no history or diagnosis of CRC. Totally, 2004 controls were recruited, and 1836 blood samples were successfully collected, with a response rate of 91.62%. A total of 680 controls (591 hospital-derived controls and 89 community-derived controls) frequency-matched to the cases on 5-year age and sex were recruited for laboratory measurement.

All participants gave written informed consent, including permission for medical records. This study was approved by the ethical committee of the School of Public Health of Sun Yat-sen University (approval number: 2019-018).

### 2.2. Data Collection

All participants completed a structured questionnaire about sociodemographic characteristics, lifestyles (e.g., smoking, passive smoking, drinking, and physical activities), family history of cancer in first-degree relatives, and an 81-item semiquantitative food frequency questionnaire (FFQ) with trained interviewers. Relevant clinical information, such as tumour node metastasis (TNM) stage and histological diagnosis were extracted from medical records. Body mass index (BMI) was calculated by dividing weight (kg) by the squared height (m^2^). Current smoking meant smoking a minimum of one cigarette per day for more than 6 consecutive months [26]. Passive smoking was defined as non-smokers being exposed to other’s tobacco smoke for a minimum of 15 min/day for the past 5 years. Drinking meant having a drink more than once a week in the last year. Working physical activities were classified by the intensity of work as (a) jobless; (b) sedentary; (c) less intensity; (d) moderate intensity; or (e) heavy intensity. Household and leisure-time physical activities were presented as the sum of metabolic equivalent task (MET) [27,28], then MET-hours/week were calculated by multiplying the hours per week spent in each activity and the MET of each activity.

Participants in this analysis completed a validated semiquantitative FFQ that included 81 food items, including food and beverages [29]. The FFQ evaluated the consumption frequencies and the mean intakes of cereal products, soya and soya products, vegetables, fruit, red and processed meat, poultry, fish and other seafood, egg, dairy products, and nuts of each participant during the 12 months before diagnosis for cases or participation in the survey for controls. The validity and reproducibility of FFQ have been demonstrated in the local population by six 3-day dietary records and the FFQ twice at a one-year interval [29], and the FFQ has been applied in previous studies [24,30,31]. The energy-adjusted Pearson correlation coefficients for the FFQ and six 3-day dietary records were 0.34 for total fat, 0.37 for SFAs, and 0.32 for monounsaturated fatty acids (MUFAs) and polyunsaturated fatty acids (PUFAs). The correlation coefficients for the reproducibility of the FFQ were 0.61 for total fat, 0.63 for SFAs and MUFAs, and 0.58 for PUFAs [24]. Daily intakes were calculated for each item based on FFQ data. According to the Chinese food composition table, total energy and nutrient intakes were calculated by multiplying the frequency of consumption of each item by the nutrient content of each item and then summing them [32].

### 2.3. Blood Collection and Measurement of Serum Fatty Acids

About 5 mL of venous fasting blood sample was collected from each participant, and none of these cases had received any treatment or surgery before blood collection. Once arrived at the laboratory, the samples were centrifuged at 3000 rpm for 10 min at 4 °C, and then serum samples were kept at −80 °C until analysis. To ensure that samples were not affected by storage conditions, serum samples from all participants were stored in the same refrigerator.

Serum levels of fatty acids were detected by gas chromatography (GC). Fatty acid methyl esters were extracted by the method previously reported [25,33]. In brief, we added 750 μL of chloroform/methanol (2:1, *v*/*v*), 200 μL of physiological saline, and 50 μL of butylated hydroxytoluene ethanol to 50 μL of serum samples and vortexed them for 1 min. The serum samples were centrifuged at 8000 rpm for 5 min at 4 °C, then the organic layer (lower chloroform layer) was transferred to a reaction vial and dried with a nitrogen-blowing apparatus to obtain the extract. Then we added 750 μL of 14% boron trifluoride-methanol solution to the extract, vortexed for 1 min, and sealed the reaction vial at 90 °C for 1.5 h. After the reaction material cooled to room temperature, we added 1 mL of hexane and 0.9% sodium chloride solution and mixed thoroughly for 1 min. The serum samples were centrifuged at 8000 rpm for 5 min at 4 °C, and then the organic layer was extracted and blown dry with nitrogen blowing. Finally, 100 μL of fatty acid methyl esters was added with hexane and transferred to a brown sample vial.

The fatty acid methyl esters were analysed by Agilent 7890A gas chromatography (Santa Clara, CA, USA) on a capillary column DB-23 (Santa Clara, CA, USA). We applied the standard product of fatty acid methyl esters as a standard. Individual fatty acid was validated using a commercial standard with known retention times. The analysers of fatty acids were completely blinded to information on participants, and peaks were calculated automatically. The serum levels of individual fatty acids were presented as the percentage (%) of total fatty acids, and we assumed undetectable levels for fatty acids values with missing data and assigned them a value of zero. To perform quality control, a quality control sample was put in every 50 samples tested, and the quality control samples were repeated every 50 samples. The intra- and inter-assay coefficients of variation ranged from 0.58 to 12.65% and 2.39 to 18.94%, respectively, which were consistent with previous results [14,34,35].

We selected the following fatty acids in this study: lauric acid (C12:0), myristic acid (C14:0), palmitic acid (C16:0), heptadecanoic acid (C17:0), stearic acid (C18:0), arachidic acid (C20:0), behenic acid (C22:0), and lignoceric acid (C24:0). We summarized the data into the following two groups: VLCSFAs (C20:0 + C22:0 + C24:0) and total SFAs (C12:0 + C14:0 + C16:0 + C17:0 + C18:0 + C20:0 + C22:0 + C24:0).

### 2.4. Statistical Analysis

All dietary intakes were adjusted for total energy intake using the residual method [36]. We compared the continuous variables between cases and control using the Wilcoxon rank-sum test, *t*-test, and the *χ*^2^-test for categorical variables. Unconditional logistic regression models were used to estimate odds ratios (ORs) and 95% confidence intervals (CIs) for the association between serum SFAs and CRC risk. Serum levels of SFAs were categorized into four levels (Quartile 1–Quartile 4, Q1–Q4) using the quartiles of each fatty acid distribution in the control group for men and women separately, and the Q1 with the lowest serum level was used as the referent group. Trends in the quartiles were tested by assigning each category to its median and using it as a continuous variable in the logistic regression model.

Potential confounders were selected according to the comparison of factors between cases and controls, combined with previous relevant studies. Variables contained in all regression models were as follows: sex, age, residence, type of job, education, marital status, income, working physical activities, household and leisure-time physical activities, smoking, passive smoking, drinking, family history of cancer in first-degree relatives, BMI, total energy intake, dietary red and processed meat intake, dietary calcium intake, dietary fibre intake, serum MUFAs, and serum PUFAs.

Stratified analysis by sex was carried out for the associations between serum SFAs and CRC risk. The interactions between sex and serum SFAs were evaluated by multiplicative models, containing the product term in the multivariable logistic regression models. Moreover, we also conducted subgroup analyses by cancer sites (colon or rectal cancer) and cancer stages (TNM stage I–II or TNM stage III–IV). Heterogeneity in the results between cancer sites or cancer stages was assessed using case-only analyses. Cancer sites or cancer stages were used as the dependent variable and serum SFAs as independent variables in the logistic regression model.

All data analyses were carried out using Stata software version 15.1 (StataCorp, College Station, TX, USA). All *p*-values were two-sided and considered significant at *p* < 0.05.

## 3. Results

Among the 680 cases, there were 350 men and 330 women. In total, 403 were diagnosed with colon cancer and 277 with rectal cancer. There were 343 cases at TNM stage I-II, and 326 cases at TNM stage III-IV. Compared to controls, more cases had a low level of education, high proportions of being married and lower income, and were likely to engage in more physical activities in work. Smoking or passive smoking was more frequent in cases than in controls. The cases tended to have a family history of cancer in first-degree relatives, consume more red and processed meat and less calcium and fibre, and have lower levels of serum PUFAs (Table 1).

Table 2 presents the percentage of serum SFAs in CRC cases and controls. The most abundant SFA in serum was palmitic acid (C16:0) in both cases and controls. Serum levels of total SFAs, lauric acid (C12:0), myristic acid (C14:0), palmitic acid (C16:0), heptadecanoic acid (C17:0), stearic acid (C18:0), and arachidic acid (C20:0) were significantly higher in cases than in controls, whereas behenic acid (C22:0), lignoceric acid (C24:0), and VLCSFAs were significantly lower among cases.

As shown in Table 3, total serum SFAs were positively associated with the risk of CRC (adjusted OR_Q4 vs. Q1_ = 2.64, 95%CI: 1.47–4.74, *p*-Trend < 0.001). However, serum VLCSFAs were inversely associated with CRC risk (adjusted OR_Q4 vs. Q1_ = 0.51, 95%CI: 0.36–0.72, *p*-Trend < 0.001). Regarding the individual SFAs, lauric acid (C12:0), myristic acid (C14:0), palmitic acid (C16:0), heptadecanoic acid (C17:0), and arachidic acid (C20:0) were positively associated with the risk of CRC (all *p*-Trend < 0.05). In contrast, behenic acid (C22:0) and lignoceric acid (C24:0) were inversely associated with CRC risk (all *p*-Trend < 0.05). However, the association of stearic acid (C18:0) with CRC risk was not statistically significant (*p*-Trend = 0.108).

Total SFAs were associated with an increased risk of CRC among women (adjusted OR _Q4 vs. Q1_ = 14.65, 95% CI: 5.60–38.32, *p*-Trend < 0.001) but not among men (*p*-Interaction < 0.001) (Table 4). Significant interactions were also observed between sex and serum myristic acid (C14:0), palmitic acid (C16:0), stearic acid (C18:0), arachidic acid (C20:0), and lignoceric acid (C24:0) on the risk of CRC (all *p*-Interaction < 0.05). In contrast, no significant interaction was observed between sex and serum lauric acid (C12:0), heptadecanoic acid (C17:0), behenic acid (C22:0), and VLCSFAs (all *p*-Interaction > 0.05). Serum VLCSFAs were inversely associated with CRC risk only in men (OR _Q4 vs. Q1_ = 0.40, 95% CI: 0.24–0.66, *p*-Trend < 0.001), but there was no significant interaction *(p*-Interaction = 0.067).

Subgroup analysis by cancer sites found no statistically significant heterogeneity in CRC risk estimates for total and individual SFAs (all *p*-Heterogeneity > 0.05) (Table 5), and similar findings were observed for subgroup analysis by cancer stages (all *p*-Heterogeneity > 0.05) (Table 6).

## 4. Discussion

This study aimed to investigate the association between serum SFAs including VLCSFAs and CRC risk. The results showed that total SFAs were positively associated with CRC risk, whereas serum VLCSFAs were inversely associated with CRC risk. Specifically, serum levels of lauric acid (C12:0), myristic acid (C14:0), palmitic acid (C16:0), heptadecanoic acid (C17:0), and arachidic acid (C20:0) were positively associated with CRC risk, while behenic acid (C22:0) and lignoceric acid (C24:0) were inversely associated with CRC risk.

To our knowledge, no published studies have investigated the association of circulating lauric acid (C12:0) with CRC risk. Our observation of the positive association between lauric acid (C12:0) and CRC risk was supported by a cellular study, which demonstrated that lauric acid induces cyclooxygenase overexpression in human tissues [37]. One study [3] found an inverse association between myristic acid (C14:0) and CRC risk. Three studies [2,15,16] did not find a significant association between myristic acid (C14:0) and CRC risk. These were contrary to our observation of a positive association between myristic acid (C14:0) and CRC risk.

Consistent with our results, two studies [2,15] found that palmitic acid (C16:0) was positively associated with CRC risk. Palmitic acid (C16:0), the predominant SFA in serum, has been found in cellular studies to increase CRC risk by upregulating the endoplasmic reticulum stress response [38] and inducing inflammatory cytokine expression [39]. In addition, palmitic acid (C16:0) has been shown to increase the expression of the β-adrenergic receptors and increase energy production in CRC, thereby promoting CRC development [40]. However, the Singapore Chinese Health Study [41] observed that palmitic acid (C16:0) was inversely associated with colon cancer risk but not associated with rectal cancer risk, and the European Prospective Investigation into Cancer and Nutrition cohort [3] found no significant association between plasma phospholipid palmitic acid (C16:0) and CRC risk.

Heptadecanoic acid (C17:0) is mainly extracted from dairy products, thus heptadecanoic acid (C17:0) can be a biomarker of dairy product consumption [8]. We found a positive association between serum heptadecanoic acid (C17:0) and CRC risk. However, two studies examining the association between heptadecanoic acid (C17:0) and CRC demonstrated a negative association [16] or no significant association [3] between erythrocyte or plasma phospholipid heptadecanoic acid (C17:0) and CRC risk. Considering the relatively low serum level of heptadecanoic acid (C17:0) in our study, further studies are needed to validate the association between heptadecanoic acid (C17:0) and CRC risk. Moreover, no significant association between serum stearic acid (C18:0) and CRC risk in our study was consistent with some previous studies [2,3,15,41]. However, two previous studies found that plasma stearic acid (C18:0) [42] or erythrocyte stearic acid (C18:0) [16] was positively associated with CRC risk.

At present, no other studies have been reported to investigate the association between circulating VLCSFAs and CRC risk. Studies examining the association of VLCSFAs with cardiovascular diseases [19,20,43], diabetes [21], and non-Hodgkin lymphoma [22] have shown beneficial findings. Two studies showed negative associations between plasma/serum lignoceric acid (C24:0) and the risk of pancreatic cancer [44] and prostate cancer [45]. Our findings of inverse associations of serum VLCSFAs, behenic acid (C22:0), and lignoceric acid (C24:0) with the risk of CRC suggest an extension of this evidence to CRC. However, a positive association between arachidic acid (C20:0) and CRC risk observed in our study are conflicting with previous studies that reported an inverse association between circulating arachidic acid (C20:0) and the risk of cardiovascular diseases [19,20,46], diabetes [21], and non-Hodgkin lymphoma [22]. This result needed to be verified. One possible mechanism for the inverse association of VLCSFAs with CRC risk is the activation of proliferator-activated receptor gamma (PPAR-γ) [47]. PPAR-γ is a ligand-activated nuclear receptor of the transcription factor superfamily and is expressed in the colon, which has been shown to significantly inhibit the proliferation and induction of CRC in vitro [48] and in vivo studies [49]. Another possible mechanism might be the involvement of VLCSFAs in the metabolism of ceramides [17], which might inhibit the development of CRC via activating the apoptosis of colon cancer cells [50,51]. However, the exact mechanism for the negative association of VLCSFAs with CRC risk remains to be elucidated.

Positive associations of total serum SFAs with CRC risk were consistent with some previous studies. A case-control study in Japan showed that total serum SFAs were positively associated with the risk of colorectal adenoma [52], a precursor lesion of CRC. In addition, total plasma phospholipid SFAs [2] or total erythrocyte SFAs [15,16] were reported to be positively associated with CRC risk. However, some studies found no significant association between total plasma phospholipid SFAs [3] or total serum SFAs [14] and CRC risk. Furthermore, our results were consistent with some studies examining the association between dietary SFAs and CRC risk. A case-control study in Korea found a positive association between dietary SFAs and CRC risk [53]. An ecological study showed that a diet high in animal fats and SFAs was associated with an increased risk of CRC [54]. The positive association of SFAs with CRC risk might be related to the activation of orphan G protein-coupled receptors (GPCRs) termed free fatty acid receptors (FFARs). FFAR4 (GPR120) is a receptor for medium- and long-chain fatty acids [55]. In vitro studies reported that the activation of FFAR4 signaling pathways promotes motile activity and angiogenesis in colon cancer cells [56]. Additionally, it has been reported that SFAs may promote CRC by affecting the sensitivity of cellular membranes to lipid peroxidation [5].

Total serum SFAs were found to be associated with an increased risk of CRC among women, but not among men, which is consistent with the Singapore Chinese Health Study [57]. One possible explanation is that palmitic acid (C16:0) activates microglia and triggers a strong inflammatory cascade response in which female microglia have a greater basal and stimulated phagocytic activity than male microglia, which may explain the different association of SFAs with CRC risk by sex [58]. Serum VLCSFAs were inversely and significantly associated with CRC risk only in men. The exact explanation for the different results in both sexes is unclear. However, plasma ferritin concentrations increased with VLCSFAs in Chinese men [59], which may lead to iron-catalysed necrosis of tumour cells and thus a negative correlation for the risk of CRC [60]. More studies are required to clarify this finding.

We found no significant heterogeneity in CRC risk estimates for total serum SFAs and individual SFAs by cancer sites. Similarly, we found no significant heterogeneity in CRC risk estimates for total serum SFAs and individual SFAs by cancer stages. No previous studies have evaluated differences between circulating SFAs and the risk of CRC by cancer sites or cancer stages. Due to the small number of cases after stratification in our study, studies with more cases are needed to verify the relationship between circulating SFAs and the risk of CRC by cancer sites or cancer stages.

To our knowledge, this is the first study to examine the association between serum SFAs and CRC risk in China. We used serum SFAs to estimate the link between SFAs and CRC risk, which can directly reflect the association between SFAs in vivo and CRC. Additionally, information on sociodemographic characteristics, lifestyles, and family history of cancer in first-degree relatives was collected and adjusted in multivariable regression models, which could reduce residual confounding as much as possible. Moreover, the levels of total serum SFAs in our study were comparable to the results of some studies among Chinese population [61,62]. A possible reason for this result is the dominant high-carbohydrate diet in the Chinese population [63], which may lead to an upregulation of de novo lipogenesis and an increase in serum palmitic acid (C16:0) [64].

There are some limitations of our study. Firstly, selection bias might occur since the CRC patients we recruited were from the same hospital. However, Sun Yat-sen University Cancer Centre is the largest cancer centre in southern China, and patients with CRC at this centre have similar clinical characteristics to patients from other hospitals in China [65]. Therefore, the results are still somewhat extrapolated. Secondly, we only had a single collection of blood samples, which may not fully represent the information before the diagnosis of CRC. However, it was reported that the changes in circulating odd-chain SFAs and even-chain SFAs over 13 years were only −0.63%/y and −0.05%/y, respectively, and there was no change in VLCSFAs over 13 years [66]. Therefore, we can assume that circulating SFAs and VLCSFAs remained largely unchanged before the diagnosis of CRC. Thirdly, GC analysis was performed at a median of 6.25 years after sample collection, and prolonged storage may affect serum fatty acids’ stability. However, previous studies did not observe significant differences in serum fatty acid composition after 8–10 years of storage at −80 °C [67]. Finally, the results of SFAs may differ from measuring different biological samples. However, previous studies found no significant differences between matched plasma and serum samples for fatty acids content in both absolute and relative values [68], which means that the results of plasma and serum fatty acids are comparable.

## 5. Conclusions

This study suggests that higher levels of total serum SFAs were associated with an elevated risk of CRC in Chinese population, while higher levels of serum VLCSFAs were associated with a lower risk. Herein, we report the first evidence that higher concentrations of serum VLCSFAs are associated with a lower risk of CRC, and this might provide new insights into the prevention of CRC. Further molecular biology studies are needed to identify the possible mechanisms of the protective roles of VLCSFAs. Since SFAs are either absorbed from dietary sources or derived from de novo fatty acid synthesis [6]; there should be different targeted dietary recommendations for different SFAs. Diets high in carbohydrates can lead to de novo synthesis of palmitic acid [64,69], and dietary palmitic acid is found mainly in animal products [7], thus we should reduce the intake of carbohydrates and animal products. In addition, a moderate reduction in the intake of dairy products should be suggested since heptadecanoic acid is mainly derived from dairy products [8]. Moreover, a moderate increase in the intake of peanuts and canola oil could also be suggested because VLCSFAs are found mainly in peanuts and canola oil. Our findings add to the growing but still limited body of evidence showing the potential differences in the health effects of different types of serum SFAs.

## Figures and Tables

**Table 1:** Sociodemographic characteristics and selected risk factors of colorectal cancer in the study population.

	Cases (n = 680)	Controls (n = 680)	*p*-Value ^1^
Age, years, mean ± SD	53.69 ± 10.71	53.25 ± 10.60	0.456
Women, n (%)	330 (48.53)	336 (49.41)	0.745
Urban, n (%)	455 (66.91)	487 (71.62)	0.060
Type of job, n (%)			0.242
Administrator/other white-collar workers	112 (16.47)	131 (19.26)	
Blue-collar worker	192 (28.24)	170 (25.00)	
Farmer/others	376 (55.29)	379 (55.74)	
Education, n (%)			<0.001
Primary school or lower	194 (28.53)	177 (26.03)	
Secondary school	200 (29.41)	166 (24.41)	
High school	174 (25.59)	157 (23.09)	
University or above	112 (16.47)	180 (26.47)	
Married, n (%)	645 (94.85)	622 (91.47)	0.013
Income, Yuan/month, n (%)			0.002
≤2000	98 (14.41)	133 (19.56)	
2001–5000	207 (30.44)	171 (25.15)	
5001–8000	208 (30.59)	176 (25.88)	
>8000	167 (24.56)	200 (29.41)	
Working physical activities, n (%)			0.019
Jobless	113 (16.62)	114 (16.76)	
Sedentary	180 (26.47)	221 (32.50)	
Less intensity	187 (27.50)	164 (24.12)	
Moderate intensity	96 (14.12)	66 (9.71)	
Heavy intensity	104 (15.29)	115 (16.91)	
Smoking, n (%)			<0.001
Never	431 (63.38)	484 (71.18)	
Current	184 (27.06)	112 (16.47)	
Former	65 (9.56)	84 (12.35)	
Passive smoking, n (%)	185 (27.21)	129 (18.97)	<0.001
Drinking, n (%)	104 (15.29)	110 (16.18)	0.655
Family history of cancer in first-degree relatives, n (%)	98 (14.41)	69 (10.15)	0.017
BMI, kg/m^2^, mean ± SD	23.28 ± 3.31	23.60 ± 3.16	0.067
Household and leisure-time physical activities, MET-hours/week, mean ± SD	32.47 ± 28.44	35.28 ± 31.09	0.081
Total energy intake, kcal/day, median (*P*_25_, *P*_75_)	1483.93 (1197.49, 1825.80)	1465.65 (1232.38, 1762.89)	0.907
Dietary red and processed meat intake, g/day, median (*P*_25_, *P*_75_) ^2^	109.20 (75.58, 144.61)	99.51 (75.41, 136.68)	0.044
Dietary calcium intake, mg/day, median (*P*_25_, *P*_75_) ^2^	361.35 (283.93, 448.67)	418.81 (329.80, 535.22)	<0.001
Dietary fibre intake, g/day, median (*P*_25_, *P*_75_) ^2^	8.27 (6.77, 10.02)	9.52 (7.60, 11.41)	<0.001
Serum MUFAs, %, median (*P*_25_, *P*_75_)	16.00 (14.55, 17.60)	15.80 (14.14, 17.66)	0.180
Serum PUFAs, %, median (*P*_25_, *P*_75_)	28.04 (25.49, 30.32)	31.00 (28.47, 33.58)	<0.001
Site, n (%)			
Colon	403 (59.26)	-	-
Rectal	277 (40.74)	-	-
TNM stage, n (%)			
Stage I–II	343 (50.44)	-	-
Stage III–IV	326 (47.94)	-	-
Unknown	11 (1.62)	-	-

Abbreviations: BMI, body mass index; MET, metabolic equivalent task; MUFAs, monounsaturated fatty acids; PUFAs: polyunsaturated fatty acids; SD, standard deviation. ^1^ Wilcoxon rank-sum test or *t*-test was used for continuous variables. *χ*^2^-test was used for categorical variables. ^2^ Dietary intakes were adjusted for total energy intake using the residual method.

**Table 2:** Percentage of serum saturated fatty acids in colorectal case and control subjects.

% of Total Fatty Acids	Cases (n = 680)		Controls (n = 680)		*p*-Value ^1^
	Mean	Median (*P*_25_, *P*_75_)	Mean	Median (*P*_25_, *P*_75_)	
Lauric acid (C12:0, %)	1.91	1.88 (1.41, 2.34)	1.67	1.66 (1.24, 2.05)	<0.001
Myristic acid (C14:0, %)	0.26	0.15 (0.11, 0.27)	0.20	0.13 (0.10, 0.19)	<0.001
Palmitic acid (C16:0, %)	34.11	33.90 (32.48, 35.53)	32.45	32.10 (30.40, 34.08)	<0.001
Heptadecanoic acid (C17:0, %)	0.63	0.37 (0.31, 0.50)	0.43	0.34 (0.29, 0.40)	<0.001
Stearic acid (C18:0, %)	16.58	16.49 (15.18, 18.04)	15.56	15.44 (14.10, 16.89)	<0.001
Arachidic acid (C20:0, %)	0.31	0.28 (0.24, 0.34)	0.27	0.26 (0.22, 0.30)	<0.001
Behenic acid (C22:0, %)	0.33	0.26 (0.19, 0.39)	0.42	0.34 (0.24, 0.48)	<0.001
Lignoceric acid (C24:0, %)	0.35	0.30 (0.23, 0.40)	0.39	0.34 (0.27, 0.45)	<0.001
VLCSFAs (%)	0.99	0.90 (0.73, 1.11)	1.08	0.98 (0.82, 1.20)	<0.001
Total SFAs (%)	51.93	51.37 (49.17, 54.38)	49.29	48.59 (45.72, 52.14)	<0.001

Abbreviations: SFAs, saturated fatty acids; VLCSFAs, very long-chain saturated fatty acids. ^1^ Wilcoxon rank-sum test was used to compare median levels of SFAs between cases and controls.

**Table 3:** Odds ratios (ORs) and 95% confidence intervals (CIs) of colorectal cancer according to quartiles of serum saturated fatty acids.

	Q1	Q2	Q3	Q4	*p*-Trend
Lauric acid (C12:0)					
Number of cases/controls	140/170	109/170	160/170	271/170	
Model 1 ^1^	1.00	0.78 (0.56–1.08)	1.14 (0.84–1.56)	1.94 (1.44–2.60)	<0.001
Model 2 ^2^	1.00	1.25 (0.85–1.83)	1.52 (1.06–2.17)	1.94 (1.38–2.74)	<0.001
Myristic acid (C14:0)					
Number of cases/controls	134/170	134/170	206/170	206/170	
Model 1 ^1^	1.00	1.00 (0.73–1.38)	1.54 (1.13–2.08)	1.54 (1.13–2.08)	<0.001
Model 2 ^2^	1.00	0.97 (0.67–1.40)	1.31 (0.92–1.86)	1.34 (0.93–1.95)	0.049
Palmitic acid (C16:0)					
Number of cases/controls	64/170	77/170	164/170	375/170	
Model 1 ^1^	1.00	1.20 (0.81–1.78)	2.56 (1.79–3.67)	5.86 (4.17–8.23)	<0.001
Model 2 ^2^	1.00	1.03 (0.66–1.60)	1.97 (1.26–3.05)	3.61 (2.14–6.10)	<0.001
Heptadecanoic acid (C17:0)					
Number of cases/controls	117/170	157/170	138/170	268/170	
Model 1 ^1^	1.00	1.34 (0.97–1.85)	1.18 (0.85–1.63)	2.29 (1.69–3.10)	<0.001
Model 2 ^2^	1.00	1.50 (1.04–2.17)	1.15 (0.79–1.67)	2.35 (1.64–3.35)	<0.001
Stearic acid (C18:0)					
Number of cases/controls	98/170	110/170	154/170	318/170	
Model 1 ^1^	1.00	1.12 (0.79–1.59)	1.57 (1.13–2.19)	3.24 (2.38–4.43)	<0.001
Model 2 ^2^	1.00	1.13 (0.75–1.71)	1.20 (0.78–1.84)	1.52 (0.92–2.52)	0.108
Arachidic acid (C20:0)					
Number of cases/controls	120/170	118/170	179/170	263/170	
Model 1 ^1^	1.00	0.98 (0.71–1.37)	1.49 (1.09–2.04)	2.19 (1.62–2.97)	<0.001
Model 2 ^2^	1.00	1.11 (0.76–1.62)	1.61 (1.12–2.31)	2.30 (1.60–3.30)	<0.001
Behenic acid (C22:0)					
Number of cases/controls	280/170	180/170	115/170	105/170	
Model 1 ^1^	1.00	0.64 (0.48–0.85)	0.41 (0.30–0.56)	0.38 (0.28–0.51)	<0.001
Model 2 ^2^	1.00	0.72 (0.52–1.00)	0.42 (0.30–0.60)	0.43 (0.30–0.60)	<0.001
Lignoceric acid (C24:0)					
Number of cases/controls	275/170	154/170	121/170	130/170	
Model 1 ^1^	1.00	0.56 (0.42–0.75)	0.44 (0.33–0.59)	0.47 (0.35–0.64)	<0.001
Model 2 ^2^	1.00	0.72 (0.52–1.00)	0.56 (0.40–0.80)	0.58 (0.41–0.81)	<0.001
VLCSFAs					
Number of cases/controls	279/170	129/170	144/170	128/170	
Model 1 ^1^	1.00	0.46 (0.34–0.62)	0.52 (0.39–0.69)	0.46 (0.34–0.62)	<0.001
Model 2 ^2^	1.00	0.59 (0.42–0.83)	0.60 (0.43–0.84)	0.51 (0.36–0.72)	<0.001
Total SFAs					
Number of cases/controls	71/170	92/170	178/170	339/170	
Model 1 ^1^	1.00	1.29 (0.88–1.88)	2.49 (1.76–3.53)	4.75 (3.40–6.62)	<0.001
Model 2 ^2^	1.00	1.12 (0.72–1.73)	1.79 (1.13–2.84)	2.64 (1.47–4.74)	<0.001

Abbreviations: Q, quartile; CIs, confidence intervals; ORs, odds ratios; SFAs, saturated fatty acids; VLCSFAs, very long-chain saturated fatty acids. ^1^ Crude OR (95% CI). ^2^ Adjusted for sex, age, residence, type of job, education, marital status, income, working physical activities, household and leisure-time physical activities, smoking, passive smoking, drinking, family history of cancer in first-degree relatives, BMI, total energy intake, dietary red and processed meat intake, dietary calcium intake, dietary fibre intake, serum MUFAs, and serum PUFAs.

**Table 4:** Odds ratios (ORs) and 95% confidence intervals (CIs) of colorectal cancer according to quartiles of serum saturated fatty acids by sex.

	Women (n = 330)			Men (n = 350)			*p*-Interaction
	Q1	Q4 ^1^	*p*-Trend	Q1	Q4 ^1^	*p*-Trend	
Lauric acid (C12:0)							
Number of cases/controls	58/84	145/84		82/86	126/86		
Adjusted OR (95% CI) ^2^	1.00	2.72 (1.63–4.56)	<0.001	1.00	1.65 (0.99–2.74)	0.061	0.138
Myristic acid (C14:0)							
Number of cases/controls	42/84	165/84		92/86	41/86		
Adjusted OR (95% CI) ^2^	1.00	4.59 (2.53–8.32)	<0.001	1.00	0.39 (0.22–0.68)	0.029	<0.001
Palmitic acid (C16:0)							
Number of cases/controls	12/84	224/84		52/86	151/86		
Adjusted OR (95% CI) ^2^	1.00	13.05 (5.52–30.85)	<0.001	1.00	1.77 (0.81–3.85)	0.065	<0.001
Heptadecanoic acid (C17:0)							
Number of cases/controls	66/84	104/84		51/86	164/86		
Adjusted OR (95% CI) ^2^	1.00	1.40 (0.83–2.37)	0.530	1.00	3.88 (2.29–6.57)	<0.001	0.056
Stearic acid (C18:0)							
Number of cases/controls	21/84	192/84		77/86	126/86		
Adjusted OR (95% CI) ^2^	1.00	5.16 (2.30–11.58)	<0.001	1.00	0.70 (0.33–1.46)	0.164	<0.001
Arachidic acid (C20:0)							
Number of cases/controls	38/84	147/84		82/86	116/86		
Adjusted OR (95% CI) ^2^	1.00	5.41 (3.02–9.68)	<0.001	1.00	1.55 (0.93–2.58)	0.053	0.006
Behenic acid (C22:0)							
Number of cases/controls	142/84	46/84		138/86	59/86		
Adjusted OR (95% CI) ^2^	1.00	0.40 (0.23–0.67)	<0.001	1.00	0.46 (0.28–0.77)	<0.001	0.963
Lignoceric acid (C24:0)							
Number of cases/controls	121/84	84/84		154/86	46/86		
Adjusted OR (95% CI) ^2^	1.00	0.88 (0.54–1.43)	0.378	1.00	0.29 (0.17–0.50)	<0.001	0.043
VLCSFAs							
Number of cases/controls	115/84	70/84		164/86	58/86		
Adjusted OR (95% CI) ^2^	1.00	0.66 (0.40–1.11)	0.203	1.00	0.40 (0.24–0.66)	<0.001	0.067
Total SFAs							
Number of cases/controls	11/84	203/84		60/86	136/86		
Adjusted OR (95% CI) ^2^	1.00	14.65 (5.60–38.32)	<0.001	1.00	0.90 (0.36–2.28)	0.876	<0.001

Abbreviations: Q, quartile; CIs, confidence intervals; ORs, odds ratios; SFAs, saturated fatty acids; VLCSFAs, very long-chain saturated fatty acids. ^1^ The table provided data for the highest quartile (Q4) and the lowest quartile (Q1) only. ^2^ Adjusted for age, residence, type of job, education, marital status, income, working physical activities, household and leisure-time physical activities, smoking, passive smoking, drinking, family history of cancer in first-degree relatives, BMI, total energy intake, dietary red and processed meat intake, dietary calcium intake, dietary fibre intake, serum MUFAs, and serum PUFAs.

**Table 5:** Odds ratios (ORs) and 95% confidence intervals (CIs) of colorectal cancer according to quartiles of serum saturated fatty acids by cancer sites.

	Colon Cancer (n = 403)				Rectal Cancer (n = 277)		*p*-Heterogeneity
	Q1	Q4 ^1^	*p*-Trend	Q1	Q4 ^1^	*p*-Trend	
Lauric acid (C12:0)							
Number of cases/controls	91/170	159/170		49/170	112/170		
Adjusted OR (95% CI) ^2^	1.00	1.82 (1.23–2.69)	0.002	1.00	2.21 (1.39–3.49)	0.001	0.454
Myristic acid (C14:0)							
Number of cases/controls	85/170	121/170		49/170	85/170		
Adjusted OR (95% CI) ^2^	1.00	1.25 (0.82–1.92)	0.190	1.00	1.59 (0.97–2.61)	0.023	0.229
Palmitic acid (C16:0)							
Number of cases/controls	35/170	239/170		29/170	136/170		
Adjusted OR (95% CI) ^2^	1.00	3.21 (1.72–5.98)	<0.001	1.00	3.73 (1.95–7.14)	<0.001	0.502
Heptadecanoic acid (C17:0)							
Number of cases/controls	73/170	166/170		44/170	102/170		
Adjusted OR (95% CI) ^2^	1.00	2.29 (1.52–3.45)	<0.001	1.00	2.32 (1.44–3.74)	0.004	0.310
Stearic acid (C18:0)							
Number of cases/controls	51/170	189/170		47/170	129/170		
Adjusted OR (95% CI) ^2^	1.00	1.44 (0.79–2.62)	0.237	1.00	2.05 (1.09–3.86)	0.030	0.098
Arachidic acid (C20:0)							
Number of cases/controls	74/170	150/170		46/170	113/170		
Adjusted OR (95% CI) ^2^	1.00	2.17 (1.43–3.31)	<0.001	1.00	2.47 (1.54–3.98)	<0.001	0.426
Behenic acid (C22:0)							
Number of cases/controls	174/170	60/170		106/170	45/170		
Adjusted OR (95% CI) ^2^	1.00	0.38 (0.25–0.58)	<0.001	1.00	0.49 (0.31–0.78)	<0.001	0.398
Lignoceric acid (C24:0)							
Number of cases/controls	170/170	77/170		105/170	53/170		
Adjusted OR (95% CI) ^2^	1.00	0.60 (0.41–0.90)	0.002	1.00	0.59 (0.38–0.92)	0.020	0.595
VLCSFAs							
Number of cases/controls	173/170	74/170		106/170	54/170		
Adjusted OR (95% CI) ^2^	1.00	0.50 (0.34–0.76)	0.001	1.00	0.57 (0.37–0.89)	0.025	0.444
Total SFAs							
Number of cases/controls	38/170	215/170		33/170	124/170		
Adjusted OR (95% CI) ^2^	1.00	2.65 (1.34–5.26)	0.001	1.00	2.93 (1.44–5.96)	0.002	0.786

Abbreviations: Q, quartile; CIs, confidence intervals; ORs, odds ratios; SFAs, saturated fatty acids; VLCSFAs, very long-chain saturated fatty acids. ^1^ The table provided data for the highest quartile (Q4) and the lowest quartile (Q1) only. ^2^ Adjusted for sex, age, residence, type of job, education, marital status, income, working physical activities, household and leisure-time physical activities, smoking, passive smoking, drinking, family history of cancer in first-degree relatives, BMI, total energy intake, dietary red and processed meat intake, dietary calcium intake, dietary fibre intake, serum MUFAs, and serum PUFAs.

**Table 6:** Odds ratios (ORs) and 95% confidence intervals (CIs) for colorectal cancer according to quartiles of serum saturated fatty acids by cancer stages.

	Stage I–II (n = 343)			Stage III–IV (n = 326)			*p*-Heterogeneity
	Q1	Q4 ^1^	*p*-Trend	Q1	Q4 ^1^	*p*-Trend	
Lauric acid (C12:0)							
Number of cases/controls	76/170	135/170		64/170	131/170		
Adjusted OR (95% CI) ^2^	1.00	1.79 (1.19–2.70)	0.004	1.00	2.02 (1.33–3.08)	0.001	0.799
Myristic acid (C14:0)							
Number of cases/controls	66/170	100/170		67/170	102/170		
Adjusted OR (95% CI) ^2^	1.00	1.44 (0.91–2.28)	0.075	1.00	1.28 (0.81–2.01)	0.092	0.845
Palmitic acid (C16:0)							
Number of cases/controls	31/170	170/170		32/170	196/170		
Adjusted OR (95% CI) ^2^	1.00	3.00 (1.55–5.78)	<0.001	1.00	4.07 (2.18–7.59)	<0.001	0.894
Heptadecanoic acid (C17:0)							
Number of cases/controls	62/170	135/170		54/170	126/170		
Adjusted OR (95% CI) ^2^	1.00	2.37 (1.54–3.65)	<0.001	1.00	2.16 (1.38–3.37)	0.002	0.831
Stearic acid (C18:0)							
Number of cases/controls	48/170	154/170		49/170	159/170		
Adjusted OR (95% CI) ^2^	1.00	2.03 (1.10–3.74)	0.015	1.00	1.36 (0.73–2.54)	0.362	0.059
Arachidic acid (C20:0)							
Number of cases/controls	63/170	129/170		56/170	128/170		
Adjusted OR (95% CI) ^2^	1.00	2.36 (1.52–3.67)	<0.001	1.00	2.28 (1.46–3.56)	<0.001	0.743
Behenic acid (C22:0)							
Number of cases/controls	141/170	49/170		135/170	55/170		
Adjusted OR (95% CI) ^2^	1.00	0.43 (0.28–0.67)	<0.001	1.00	0.41 (0.26–0.63)	<0.001	0.923
Lignoceric acid (C24:0)							
Number of cases/controls	136/170	63/170		135/170	65/170		
Adjusted OR (95% CI) ^2^	1.00	0.58 (0.38–0.88)	0.005	1.00	0.55 (0.36–0.84)	0.002	0.836
VLCSFAs							
Number of cases/controls	141/170	59/170		134/170	66/170		
Adjusted OR (95% CI) ^2^	1.00	0.53 (0.35–0.81)	0.001	1.00	0.51 (0.33–0.77)	0.006	0.318
Total SFAs							
Number of cases/controls	35/170	159/170		35/170	173/170		
Adjusted OR (95% CI) ^2^	1.00	3.12 (1.54–6.32)	<0.001	1.00	2.43 (1.20–4.90)	0.007	0.283

Abbreviations: Q, quartile; CIs, confidence intervals; ORs, odds ratios; SFAs, saturated fatty acids; VLCSFAs, very long-chain saturated fatty acids. ^1^ The table provided data for the highest quartile (Q4) and the lowest quartile (Q1) only. ^2^ Adjusted for sex, age, residence, type of job, education, marital status, income, working physical activities, household and leisure-time physical activities, smoking, passive smoking, drinking, family history of cancer in first-degree relatives, BMI, total energy intake, dietary red and processed meat intake, dietary calcium intake, dietary fibre intake, serum MUFAs, and serum PUFAs.

## Data Availability

This is a case-control study, where all anonymized data were obtained from secured electronic medical records. Data sharing is not applicable to this article. The authenticity of this article has been validated by uploading the key raw data onto the Research Data Deposit public platform (www.researchdata.org.cn (accessed on 4 January 2023)), with the approval RDD number RDDA2023386891.
